# Supplementary material for: High CD169 Monocyte/Lymphocyte Ratio Reflects Immunophenotype Disruption and Oxygen Need in COVID-19 Patients
Source: Pathogens. 2021 Dec 18;10(12):1639. doi: 10.3390/pathogens10121639 (PMC8715749; doi:10.3390/pathogens10121639)
Supplement: Supplementary file 1 [file pathogens-10-01639-s001.zip › Table S1.pdf]

Table S1: ROC curve for CD169 RMFI and CD169 in monocytes in COV with respect to HD. The area under ROC curve (AUC) is indicated as well as the sensitivity and specificity of the test.

COV vs HD	AUC	Sensitivity (%)	Specificity (%)
**CD169 RMFI**	0.925 $p<0.001$	97	92
**CD169 MFI**	1.000 $p=0.023$	100	83
