# Supplementary figures and images for: High CD169 Monocyte/Lymphocyte Ratio Reflects Immunophenotype Disruption and Oxygen Need in COVID-19 Patients
Source: Pathogens. 2021 Dec 18;10(12):1639. doi: 10.3390/pathogens10121639 (PMC8715749; doi:10.3390/pathogens10121639)

A)

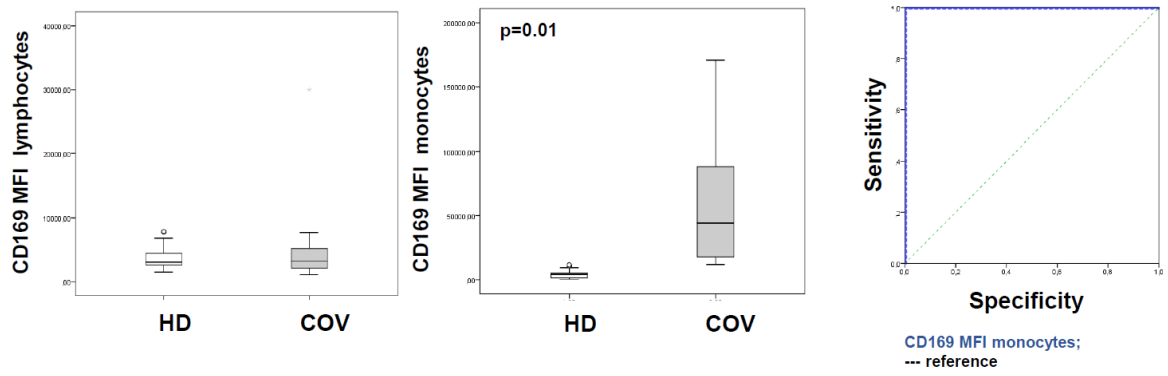

B)

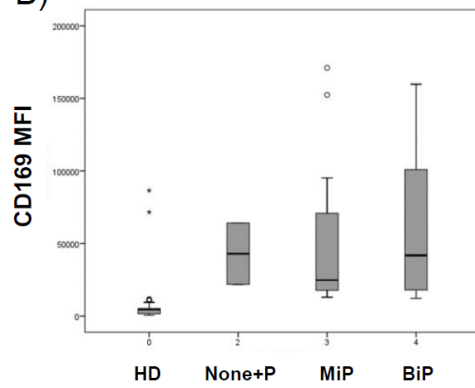

C)

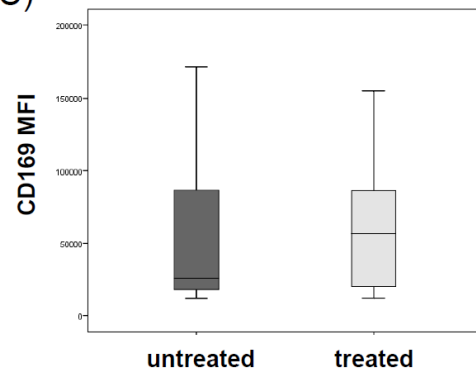

Supplement: Supplementary file 1 [file pathogens-10-01639-s001.zip › Figure S1.pdf]

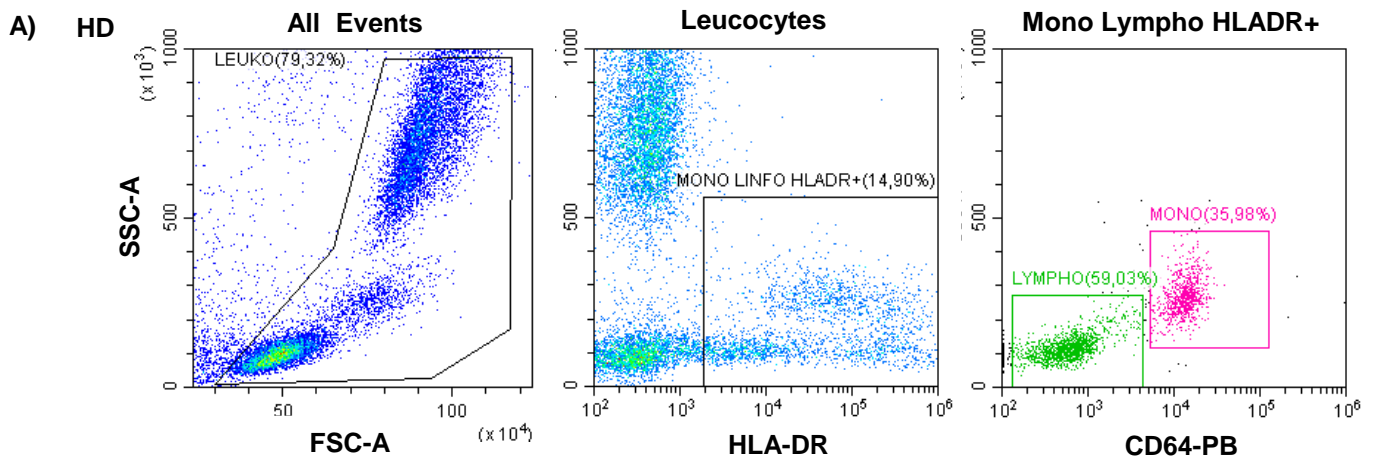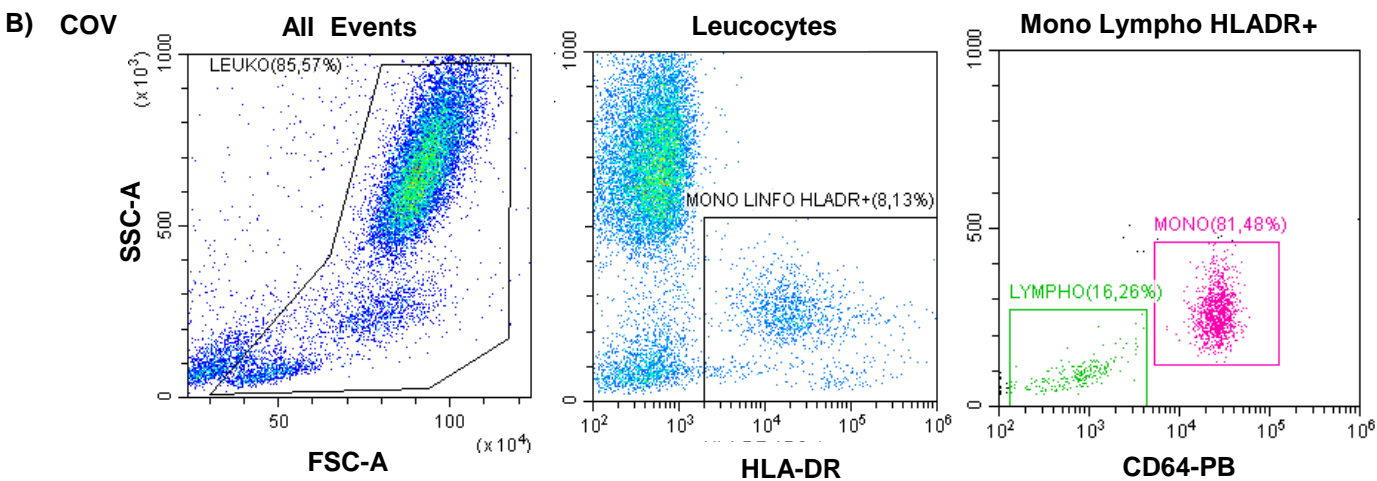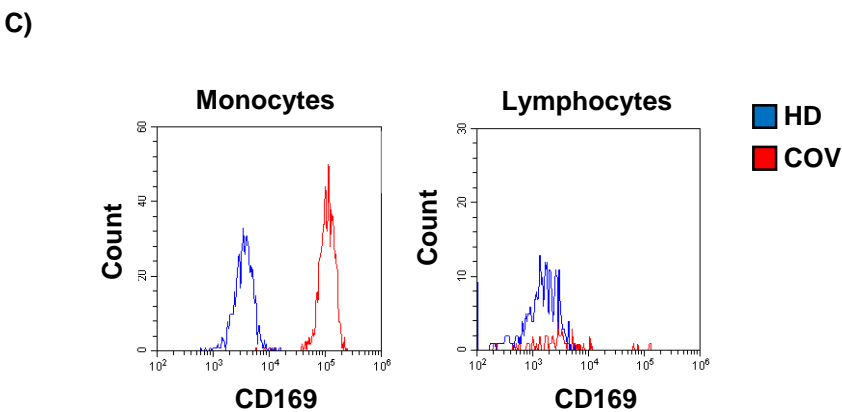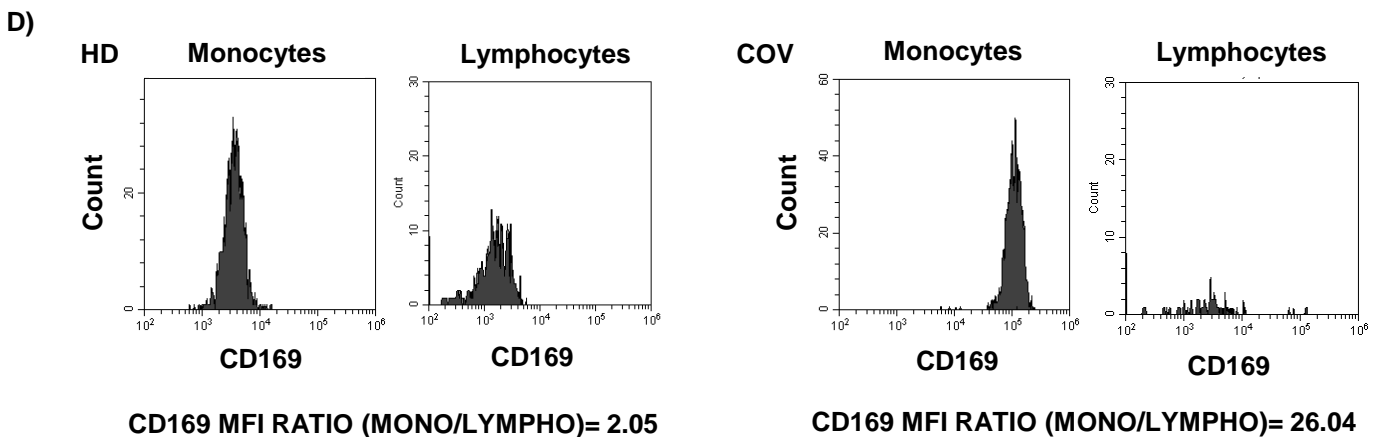

Supplement: Supplementary file 1 [file pathogens-10-01639-s001.zip › Figure S2.pdf]
